# Gene function analysis and underlying mechanism of esophagus cancer based on microarray gene expression profiling

**DOI:** 10.18632/oncotarget.22160

**Published:** 2017-10-30

**Authors:** Ying Yue, Mengjia Song, Yamin Qiao, Pupu Li, Yiqiang Yuan, Jingyao Lian, Suying Wang, Yi Zhang

**Affiliations:** ^1^ Biotherapy Center, The First Affiliated Hospital of Zhengzhou University, Zhengzhou, Henan 450052, China; ^2^ Department of Oncology, The First Affiliated Hospital of Zhengzhou University, Zhengzhou, Henan 450052, China; ^3^ The No.7. People's Hospital of Zhengzhou, Zhengzhou, Henan 450016, China; ^4^ Clinical Laboratory, Hebi People's Hospital, Hebi 458030, China; ^5^ School of Life Sciences, Zhengzhou University, Zhengzhou, Henan 450001, China; ^6^ Key Laboratory for Tumor Immunology and Biotherapy of Henan Province, Zhengzhou, Henan 450052, China

**Keywords:** esophagus cancer, gene function analysis, microarray gene expression profiling

## Abstract

Esophageal cancer (EC) is one of the most common digestive malignant tumors worldwide. Over the past decades, there have been minimal improvements in outcomes for patients with EC. New targets and novel therapies are needed to improve outcomes for these patients. This study aimed to explore the molecular mechanisms of EC by integrated bioinformatic analyses of the feature genes associated with EC and correlative gene functions which can distinguish cancerous tissues from non-cancerous tissues. Gene expression profile GSE20347 was downloaded from Gene Expression Omnibus (GEO) database, including 17 EC samples and their paired adjacent non-cancerous samples. The differentially expressed genes (DEGs) between EC and normal specimens were identified and then applied to analyze the GO enrichment on gene functions and KEGG pathways. Corresponding Pathway Relation Network (Pathway-net) and Gene Signal Network (signal-net) of DEGs were established based on the data collected from GCBI datasets. The results showed that DEGs mainly participated in the process of cell adhesion, cell proliferation, survival, invasion, metastasis and angiogenesis. Aberrant expression of PTK2, MAPK signaling pathway, PI3K-Akt signaling pathway, p53 signaling pathway and MET were closely associated with EC carcinogenesis. Importantly, Interleukin 8 (IL8) and C-X-C chemokine receptor type 7 (CXCR-7) were predicted to be significantly related to EC. These findings were further validated by analyzing both TCGA database and our clinical samples of EC. Our discovery provides a registry of genes and pathways that are disrupted in EC, which has the potential to be used in clinic for diagnosis and target therapy of EC in future.

## INTRODUCTION

Esophageal cancer (EC) is one of the most common digestive malignant tumors and the sixth main cause of cancer-related death worldwide [1, 2]. Despite incremental advances in diagnostics and therapeutics, esophageal cancer still carries a poor prognosis and the 5-year survival rate of advanced EC patients is less than 15%[3]. Studies have reported that the prognosis for patients was varied because of the heterogeneous nature of EC. As with other malignant tumors, the occurrence and development of EC is a complex process with multi-step and multiple factors and the molecular pathogenesis has not been fully elucidated [4–6]. Thus, a compelling need exists to extensively identify genomic abnormalities underlying EC, for elucidating its molecular basis and guiding the development of effective targeted therapies and prevention.

With the advent of next-generation sequencing technologies, transcriptome sequencing (RNA-Seq) has become a powerful tool for comprehensive characterization of the whole transcriptome at gene and exon levels and with a unique ability to identify genetic variations, novel splicing variants, and transcripts at high resolution and efficiency [7–9]. Transcriptome is a set of all RNA transcripts including mRNA, tRNA, rRNA and non-coding RNA such as miRNA produced in one or a population of certain type of cells [10]. Unlike genome, which is roughly fixed for a certain type of cells, the transcriptome can vary with external environmental condition and it is considered to be highly dynamic. When the cells suffer different physiologic or pathologic stimuli, their transcriptome will change dramatically [11]. By RNA sequencing analysis, several genes, long noncoding RNA (lncRNA) and microRNA (miRNA) were reported to function as biomarkers and indicators of prognosis of EC [12]. The down/over expression of protein coding genes such as MRP14 gene [13], PTK6 [14], elongation factor 1 gamma [15] in tumor tissues may provide preoperative useful information for predicting the aggressiveness of tumors. In addition, some studies showed that miRNA-211 [16], miRNA-143 [17], miRNA-183 [18], and lncRNA CASC9 [19], HOTAIR [20], POU3F3 [21] contributed to the development of EC via a variety of mechanisms. Transcriptomic changes inherit from genomic information and take place before the proteomic level. Understanding of this crucial stage of genomic information process is of most importance for us to unveal the mechanisms of tumorigenesis. In our study, we purposed the idea of dynamic transcriptome and put it forward to the study of EC transcriptomics and establishment of gene expression regulation network.

## RESULTS

### Data preprocessing

**Figure 1:**
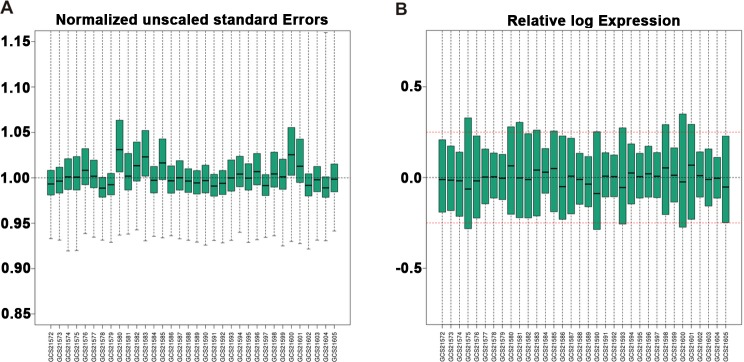
Box plots of gene expression data of 17 EC and 17 normal tissues after standardization **(A)** NUSE and **(B)** RLE of these data after standardization are within an acceptable range. Black lines in the boxes represent medians. Red lines in the boxes represent the standard criterion of RLE. NUSE, Normalized Unscaled Standard Errors. RLE, Relative Log Expression. EC, Esophageal cancer.

Gene expression profile GSE20347 was downloaded from Gene Expression Omnibus (GEO) database, including 17 EC samples and their paired adjacent non-cancerous samples. Quality control of gene expression data was performed using gene-specific probe. Normalized Unscaled Standard Errors (NUSE) and Relative Log Expression (RLE) of these data after standardization were shown in Figure 1A and Figure 1B. All the NUSE and RLE in the figure were within an acceptable range, suggesting that the results of subsequent analysis were reliable. The expression profile data were firstly preprocessed and then analyzed by GCBI online platform. Total of 22278 genes were screened. And the entire bioinformatic workflow was shown in Supplementary Figure 1.

### Differentially expressed genes (DEGs) screening

**Figure 2:**
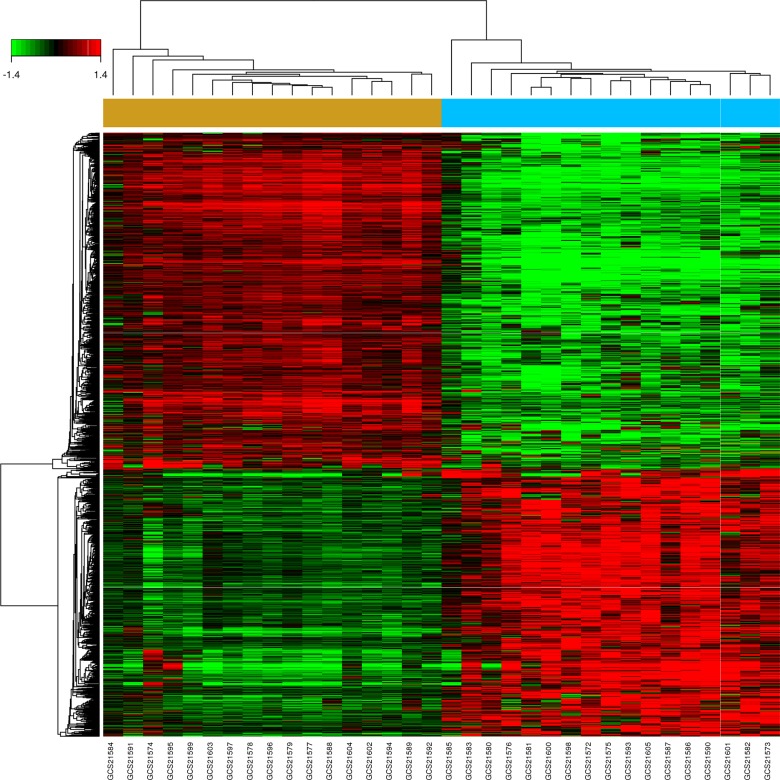
Hierarchical cluster dendrogram of DEGs The horizontal axis represents sample names. The brown part represents normal samples and the blue part represents EC samples. The left vertical axis shows clusters of DEGs, and the above horizontal axis shows clusters of samples. Red represents up-regulated genes and green represents down-regulated genes.

A total of 1348 DEGs, 623 up-regulated and 725 down-regulated, were identified in 17 EC samples compared with their paired normal tissues (P value < 0.05 and false discovery rate (FDR) < 0.05). Hierarchy cluster analysis showed that the 17 EC samples distributed in EC sample cluster and the 17 paired normal samples in normal sample cluster (Figure 2), indicating that grouping procedure was reasonable and applicable to further analysis. The top 20 significantly up-regulated DEGs and down-regulated DEGs were shown in Table 1 according to Fold Change (FC). We could concluded that the overexpression of MMP family members (MMP1, MMP12, MMP10), SSP1, collagen family members (COL11A1, COL1A1, COL1A2), SULF1, CDH1, INHBA, VCAN as well as APOBEC3B played a vital role in the process of EC occurrence and development. In addition, the data from TCGA was used for further validations. The different expressions between cancer and paired normal tissues of the above-described overexpressed genes were still very significant in TCGA database. In Supplementary Figure 2A and 2B, we have listed some of the results.

**Table 1:** The top20 significant up-regulated and down-regulated DEGs involved in EC according to fold change

Group	Gene Symbol	Gene Description	Fold Change	p-value
Upregulated genes	MMP1	matrix metallopeptidase 1 (interstitial collagenase)	33.299251	0.000199
	SPP1	secreted phosphoprotein 1	24.812088	0.000105
	COL11A1	collagen, type XI, alpha 1	20.854271	0.000211
	NTS	neurotensin	15.378321	0.007374
	POSTN	periostin, osteoblast specific factor	13.764033	0.000511
	ANO1	anoctamin 1, calcium activated chloride channel	13.329332	0.000648
	MMP12	matrix metallopeptidase 12 (macrophage elastase)	12.706198	0.000068
	VCAN	versican	10.73413	0.000315
	IL8	interleukin 8	10.236861	0.000172
	COL1A2	collagen, type I, alpha 2	9.234818	0.001952
	COL1A1	collagen, type I, alpha 1	9.214333	0.001832
	JUP	junction plakoglobin	8.955021	0.001329
	MMP10	matrix metallopeptidase 10 (stromelysin 2)	8.592152	0.002876
	AKR1C1	aldo-keto reductase family 1, member C1	8.546899	0.004894
	SULF1	sulfatase 1	7.965726	0.000175
	ZIC1	Zic family member 1	7.84641	0.000662
	APOBEC3B	apolipoprotein B mRNA editing enzyme, catalytic polypeptide-like 3B	7.596131	0.000517
	CDH11	cadherin 11, type 2, OB-cadherin (osteoblast)	7.206682	0.000612
	INHBA	inhibin, beta A	7.125975	0.001156
Downregulated genes	CRISP3	cysteine-rich secretory protein 3	−145.091859	0.000046
	CRNN	cornulin	−84.394306	0.000063
	MAL	mal, T-cell differentiation protein	−57.860795	0.000076
	CRCT1	cysteine-rich C-terminal 1	−49.533869	0.000079
	TGM3	transglutaminase 3 (E polypeptide, protein-glutamine-gamma-glutamyltransferase)	−47.423993	0.000069
	SCEL	sciellin	−41.055178	0.00006
	CLCA4	chloride channel accessory 4	−40.459777	0.000113
	CLIC3	chloride intracellular channel 3	−40.15493	0.000051
	KRT4	keratin 4	−33.981117	0.000344
	SLURP1	secreted LY6/PLAUR domain containing 1	−32.55029	0.000047
	SPINK5	serine peptidase inhibitor, Kazal type 5	−32.391855	0.000058
	TMPRSS11E	transmembrane protease, serine 11E	−24.154135	0.000129
	ENDOU	endonuclease, polyU-specific	−23.847101	0.000045
	HPGD	hydroxyprostaglandin dehydrogenase 15-(NAD)	−20.91442	0.000089
	KLK13	kallikrein-related peptidase 13	−20.206265	0.00009
	CEACAM7	carcinoembryonic antigen-related cell adhesion molecule 7	−19.141801	0.000169
	FLG	filaggrin	−19.015206	0.000089
	EREG	epiregulin	−18.937187	0.000046
	CXCR2	chemokine (C-X-C motif) receptor 2	−17.054968	0.000046
	PPP1R3C	protein phosphatase 1, regulatory subunit 3C	−17.054788	0.000047
	KRT13	keratin 13	−16.592631	0.001601
	CYP4B1	cytochrome P450, family 4, subfamily B, polypeptide 1	−16.56291	0.000102

Interleukin 8 (IL8) as the first significantly up-regulated chemokine, was overexpressed for more than 10 times. And C-X-C chemokine receptor type 7 (CXCR-7), as the first significantly up-regulated chemokine receptor, was overexpressed for more than 5 times in EC. Similarly, we have verified in the TCGA database. We found that the different expressions between cancer and paired normal tissues of IL8 and CXCR7 were still significant. Furthermore, the expression of IL8 and CXCR7 negatively correlated to progression-free survival (PFS). And the expression of CXCR7 was also related to the overall survival (OS) of the patient, whereas IL8 expression was not associated with OS (Supplementary Figure 3). The significant down-regulation of some genes was also determined in EC samples, such as CRISP3, CRNN, MAL, CRCT1, TGM3 and SCEL.

### Functional enrichment analysis

**Figure 3:**
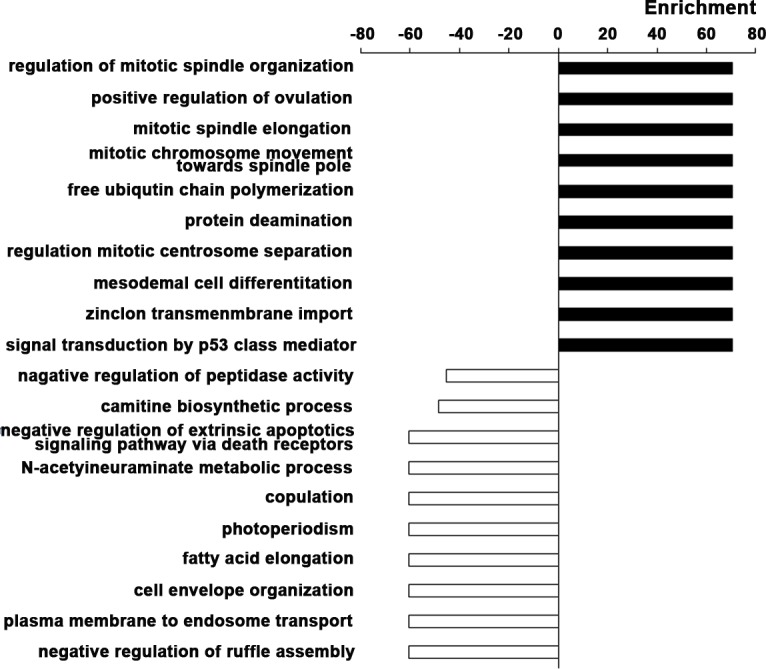
Gene Ontology enrichment analyses The top 10 significantly up-regulated and down-regulated GO categories in EC based on the functional enrichment. The horizontal axis represents the enrichment score of DEGs. The vertical axis represents the GO categories.

In order to make functional interpretation for the gene expression changes, we performed GO analysis based on Fisher exact test. Functional enrichment analysis was performed for all DEGs. The result revealed a total of 446 significant GO categories of up-regulated genes and a total of 173 significant functional GO categories of down-regulated genes (P value < 0.05 and FDR < 0.05). The top 10 significantly up-regulated and down-regulated GO categories were shown based on the functional enrichment (Figure 3). It was concluded that the up-regulated DEGs were mainly involved in the regulation of cell proliferation, such as the process of mitotic and signal transduction of p53, a key molecule of cell cycle. Significant overexpression of positive regulation of ovulation, free ubiquitin chain polymerization, protein deamination, mesodermal cell differentiation, zinc ion transmembrane import were also associated with EC. As for the down-regulated DEGs, negative regulation of peptidase activity, carnitine biosynthetic process, negative regulation of extrinsic apoptotic signaling pathway via death domain receptors, N-acetylneuraminate metabolic process, copulation, photoperiodism, fatty acid elongation, cell envelope organization, plasma membrane to endosome transport, negative regulation of ruffle assembly were closely related to the pathogenesis of EC.

**Figure 4:**
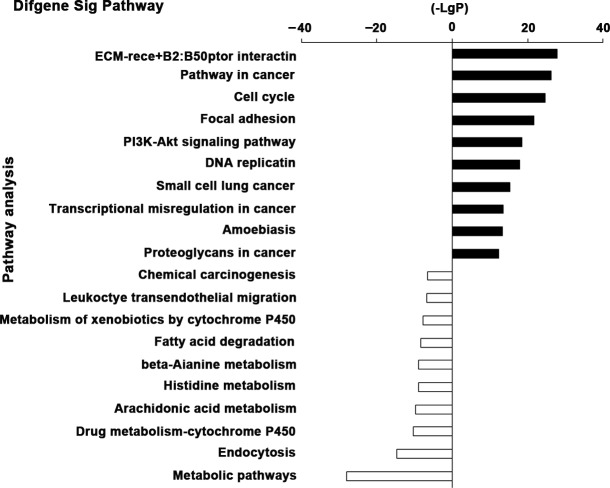
The top 10 significant pathways of the DEGs The top 10 significantly changed pathways of up-regulated and down-regulated DEGs were shown respectively. The vertical axis is the pathway categories, and the horizontal axis is the negative logarithm of the *P* value (−Lg*P*) of pathways.

Pathway analysis was constructed to identify the key pathways that DEGs were involved in. As shown in the result, the up-regulated DEGs were enriched in a total of 74 significant pathways and the down-regulated DEGs were enriched in a total of 103 significant pathways (*P* value < 0.05 and FDR < 0.05). The top 10 significant pathways of up-regulated and down-regulated DEGs were shown in Figure 4 according to the negative logarithm of the *P* value (−Lg*P*). Dramatically up-regulated pathways that DEGs participated in were extracellular matrix receptor (ECM-receptor) interaction, pathways in cancer, cell cycle, focal adhesion, PI3K-Akt signaling pathway and more, indicating that aberrant cell adhesion and carcinogenic pathways played an important role in EC carcinogenesis. Pathways dramatically altered in down-regulated genes demonstrated that the disruption of metabolism pathways, xenobiotics by cytochrome P450, fatty acid degradation and beta-Alanine metabolism were crucial factors of EC progression. Additionally, the down-regulation of endocytosis, leukocyte transendothelial migration and chemical carcinogenesis were also closely related to EC.

### Pathway relation network (pathway-net) analysis of the significant pathways

**Figure 5:**
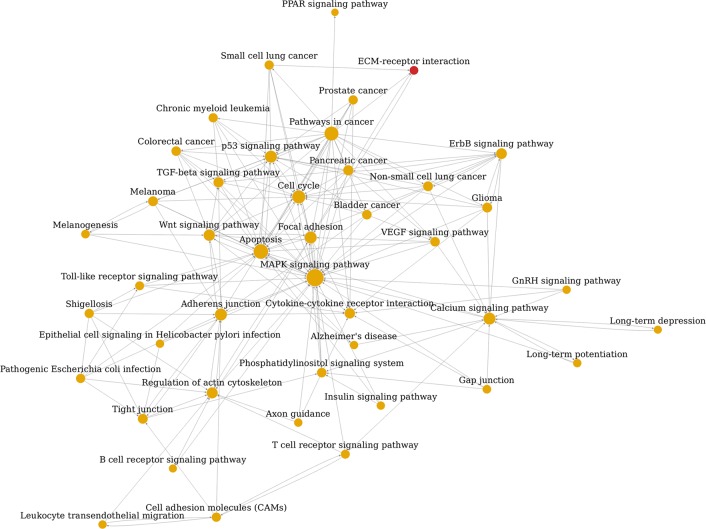
Pathway network (Path-net) analysis Significantly changed pathways are connected in a Path-net to show the relationship between these pathways. Each pathway in the network is measured by counting the upstream and downstream pathways, which are shown as in-degree, out-degree or degree, respectively. A higher degree of pathway indicates that it regulates or is regulated by other pathways, implying a more important role in the signaling network. The yellow circle represents the both up-regulated and down-regulated pathway and the red represents the up-regulated pathway. The lines show the interaction between pathways.

To systematically understand the central pathways involved in EC pathogenesis and the interconnection of them, we constructed the pathway-net of the significant DEGs according to the theory and relationship provided by KEGG pathway database. As shown in Figure 5, the main pathways implicated in EC were MAPK signaling pathway, apoptosis, pathways in cancer, cell cycle, calcium signaling pathway, p53 signaling pathway, focal adhesion, adherens junction, wnt signaling pathway and VEGF signaling pathway. Degree number of pathways in the network represented their interconnection complexity with other pathways. The degree numbers of top 10 significant pathways were shown in Table 2. It was noticed that MAPK signaling pathway was involved with others most extensively.

**Table 2:** The top10 significant pathways identified by pathway-net analysis of DEGs associated with EC according to degree number

Pathway Name	Outdegree	Indegree	Degree
MAPK signaling pathway	5	28	33
Apoptosis	3	21	24
Pathways in cancer	22	0	22
Cell cycle	2	17	19
Calcium signaling pathway	5	10	15
p53 signaling pathway	2	13	15
Focal adhesion	8	7	15
Adherens junction	6	9	15
Wnt signaling pathway	7	6	13
Glycolysis / Gluconeogenesis	2	10	12

### Gene signal network (signal-net) analysis of the significant DEGs

**Figure 6:**
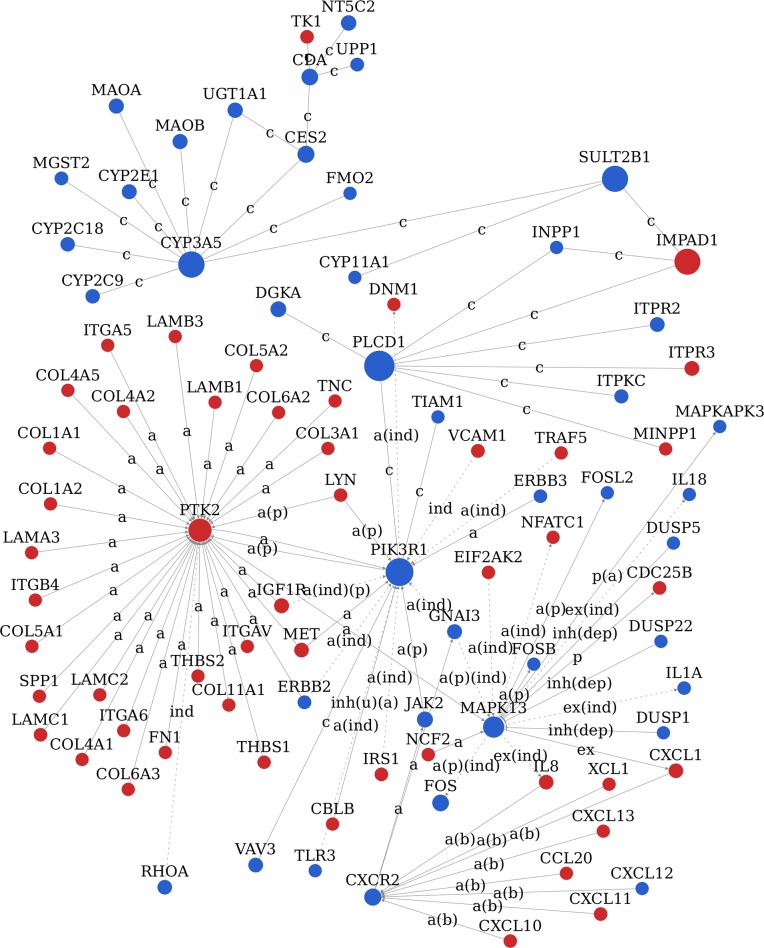
Signal network (Signal-net) analysis The red circle represents the up-regulated DEGs and the blue circles down-regulated DEGs. The area of the circle represents the betweenness. Interaction between the genes is shown as a activation, a(b) activation(binding/association), a(ind) activation (indirect effect), a(p) activation(phosphorylation), a(ind)(p) activation(indirect effect)(phosphorylation), a(p)(ind) activation(phosphorylation)(indirect effect), c compound, ex expression, ex(ind) expression(indirect effect), ind indirect effect, inh(dep) inhibition(dephosphorylation), inh(u)(a) inhibition(ubiquitination)(activation), p(a) phosphorylation(activation).

In order to clarify the interaction between different gene products, signal-net of the significant DEGs was established based on the KEGG database and the theory of network biology (Figure 6). Signal-net could break through the limit of acquiring the interactions of between genes in single pathway and obtain some protein's upstream or downstream proteins through the whole KEGG-Pathway database. The network provided us with the key drivers of EC, including the disruption of PLCD1, PIK3R1, SULT2B1, IMPAD1, CYP3A5, PTK2, MAPK13, GATM, SHMT1, CXCR2 and MET. The top 10 significant DEGs identified by signal-net analysis were shown in Table 3 according to betweenness value.

**Table 3:** The top10 significant DEGs identified by signal-net analysis in EC according to betweenness value

Gene Symbol	Gene Feature	Betweenness	Indegree	Outdegree
PLCD1	down	10047.3333	7	7
PIK3R1	down	9022.6167	16	5
SULT2B1	down	7918	3	3
IMPAD1	up	7889	3	3
CYP3A5	down	7780.8183	9	9
PTK2	up	6435.85	31	2
MAPK13	down	5025.5	6	10
GATM	down	2820.75	4	4
SHMT1	down	2627	3	3
CXCR2	down	2377.2984	8	2

### Validation of microarray data in clinical samples by real time polymerase chain reaction (RT-PCR) and immunohistochemistry (IHC)

**Figure 7:**
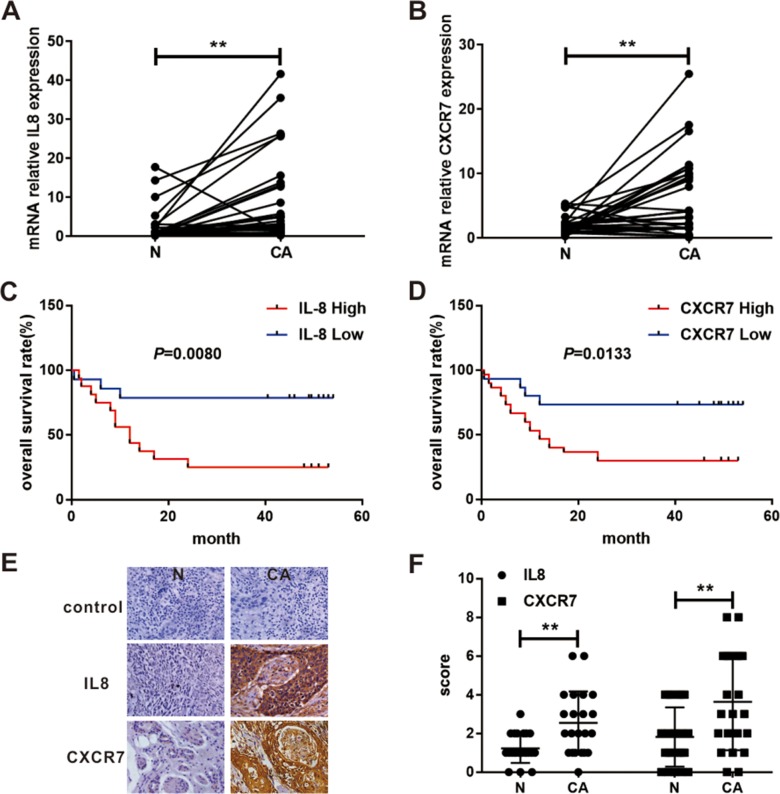
Expression and survival significance of IL8 and CXCR7 in patients with EC **(A)** IL8 mRNA relative expression in cancerous and their paired adjacent non-cancerous tissues in 30 patients with EC were analyzed by RT-PCR. **(B)** CXCR7 mRNA relative expression in cancerous and their paired adjacent normal tissues in 30 patients with EC were analyzed by RT-PCR. Kaplan-Meier survival curve for 30 EC patients with high (n=15) and low (n=15) IL8 **(C)** and CXCR7 **(D)** expression (RT-PCR analysis). **(E)** IL8 and CXCR7 expression in cancerous and paired adjacent normal tissue of EC were analyzed by IHC. **(F)** The final immunoreactivity score of IL8 and CXCR7 expression in 22 paired specimens was shown as statistical graph. (^**^P < 0.01; N, non-cancerous tissue; CA, cancerous tissue).

As shown in Table 1 that IL8 and CXCR7 were not the most obvious DEGs. However, IL8 and CXCR7 were the most significant chemokines and chemokine receptors that were up-regulated in our results. Numerous studies have shown that chemokines and chemokine receptors played an important role in the development of tumors [29]. We believed that the study of high-level CXCR7 and IL8 in EC would also be of great significance. And the upregulation and significance of IL8 and CXCR7 have been validated in the TCGA database (Supplementary Figure 3). To further validate these findings in our system, we analyzed the expression of IL8 and CXCR7 in 30 patients with EC at mRNA level. The PCR results showed that the expression of IL8 and CXCR7 was higher in cancerous tissues compared to normal tissues (Figure 7A, 7B). Moreover, the mRNA expression of IL8 and CXCR7 negatively correlated to the overall survival rate of EC patients (Figure 7C, 7D). In addition, higher expression of IL8 and CXCR7 in cancerous tissues was identified by IHC (Figure 7E, 7F). These findings indicated that IL8 and CXCR7 might be the potential biomarkers of EC and could be used as therapeutic targets for patients with EC.

## DISCUSSION

EC is one of the most common digestive malignant tumors and the sixth main cause of cancer-related death worldwide [1, 2], the development of which is the process that a multi-step and multi-gene involved in. A variety of studies have analyzed the global gene expression changes in malignant and paired normal parts of esophageal tissues by genome and transcriptome microarray [30–33]. In 2011, Su et al analyzed the DEGs in tumor subtypes of EC and identified their risk factors [32]. However, the molecular pathogenesis of EC has not been fully elucidated [4–6]. A compelling need exists to extensively identify genomic abnormalities underlying ESCC, for elucidating its molecular basis and guiding the development of effective targeted therapies and prevention. RNA-Seq has become a powerful tool for comprehensive characterization of the whole transcriptome at gene and exon levels and with a unique ability to identify genetic variations, novel splicing variants, and transcripts at high resolution and efficiency [7–9]. In this study, we focus on investigating the potential targets of EC by analyzing the DEGs between malignant and normal tissues and their functions and regulatory pathways they participated in. We analyzed the different gene expression of 17 EC samples and their paired adjacent non-cancerous sample and further validated the findings using TCGA database. The results suggested that EC progression was strongly associated with (1) cell proliferation, survival, invasion, metastasis and angiogenesis (MAPK signaling pathway, cell cycle, wnt signaling pathway, VEGF-signaling pathway, MMP1, SPP1, COL11A1), (2) cell adhesion (focal adhesion, adhesion junction, calcium signaling pathway, PTK2),(3) the imbalance of oncogene and cancer suppressor gene (p53 signaling pathway, pathway in cancer, MET, PLCD1) as well as(4) the participation of the immune system (IL8, CXCR7).

Tumor cell proliferation, survival, migration, invasion and angiogenesis were important to maintain the malignant phenotype for a variety of cancers. Our results of pathway analysis and pathway-net indicated that MAPK and PI3K-Akt signaling pathway were the key pathways involved in EC carcinogenesis, which has been revealed to increase esophageal cancer cell growth, proliferation, migration and invasion [34–36]. In accord with previous studies, cell cycle and wnt signaling pathway were significantly up-regulated in EC, which was strongly associated with the property of cell proliferation and self-renewal [37, 38]. Furthermore, it has been identified that the expression of MMP1, SPP1 and COL11A1 positively correlated with poor prognosis of patents with EC [39–43]. Our data further validated their high expression in EC. In addition, we also found VEGF signaling pathway was significantly overexpressed in EC, suggesting that angiogenesis was extensively implicated in carcinogenesis. Additionally, the imbalance of oncogene and cancer suppressor gene expression was an crucial mechanism of EC progression, such as the overexpression of p53 signaling pathway and MET and the down-regulation of PLCD1, the encoded protein of which functions as a tumor suppressor in several types of cancer [44–46]. Importantly, several studies about genomic landscape have validated that frequent disruption of MAPK signaling pathway, PI3K-Akt signaling pathway, cell cycle, wnt signaling pathway and MET were strongly involved in ESCC [12, 14, 47]. Taken together, these up-regulated pathways and genes involved in tumor cell proliferation, survival, invasion and angiogenesis might be taken as diagnostic biomarkers and potential therapeutic targets for EC.

As one mechanism of tumor invasion and metastasis, aberrant adhesion of tumor cells to extracellular matrix and other cell types has been elucidated to be a typical phenotype of various malignances [48–50]. In our study, both pathway analysis and pathway-net identified that focal adhesion, adhesion junction and calcium signaling pathway were highly expressed in EC. Signal-net showed that PTK2, a gene concentrated in the focal adhesions, was the most important up-regulated gene in EC. Activation of PTK2 might be an important step in tumor cell growth and interactions with the ECM [51–53]. In addition, amounts of genes encoding other adhesion molecules, such as LAMB3, LAMC1, COL1A1, COL6A2, ITGAV, THBS1 and more, were also highly expressed in EC. Collectively, upregulation of cell adhesion was essential for EC carcinogenesis and might be a diagnostic marker of EC.

Finally, immune system malfunction was known to play a prominent role in the tumorigenesis of many malignancies. Our results demonstrated that IL8 and CXCR7, the important members of chemokines and chemokine receptors family, were dramatically up-regulated and CXCR2 was dramatically down-regulated. Previous studies validated that IL8 promoted the esophageal carcinoma cell invasiveness, metastasis and angiogenesis [54–56]. IL8 expression positively correlated with tumor angiogenesis and survival rates of esophageal squamous cell carcinoma (ESCC) patients [56]. IL8 is a kind of both cytokine and chemokine, and our lab has been exploring its immune-suppressive role in EC (unpublished). It reminds that IL8 may be a novel biomarker and target of EC patients. It has been reported that CXCR7 is high-expression in ESCC [57] and associated with poor recurrence-free survival and cause-specific survival (CSS) in patients with ESCC [58]. CXCR7 could markedly promote esophageal cancer cell proliferation, migration and invasion as well as tumor growth [59, 60]. Our lab also confirmed high expression of CXCR7 was closely related to malignant biological behavior and stemness of esophageal cancer cell (unpublished). It could be concluded that CXCR7 represented a potentially therapeutic target for EC. Similarly, CXCR2 was reported to participate in increasing cell viability, chemotaxis and invasion and decreasing apoptotic rate [60]. Instead of up-regulated, the results of our study indicated that CXCR2 was down-regulated in EC. Therefore, the exact effect of CXCR2 on EC and corresponding mechanism need to be further investigated. Other chemokines of the CXC subfamily were also aberrant expression in EC, including upregulation of CXCL1, CXCL10, CXCL11, CXCL13, and down-regulation of CXCL20. These chemokines not only regulated leucocyte infiltration but also participated in tumor cell invasion, migration, adhesion of ECM [61, 62]. Therefore, the down-regulation of leukocyte transendothelial migration revealed by pathway analysis might be caused by the imbalanced expression of chemokines in EC. Furthermore, the results of TCGA database and our clinical samples further supported our view which showed the significantly different expressions between cancerous and normal tissues of IL8 and CXCR7 and the positive correlation of them with the poor prognosis of patients with EC (Supplementary Figure 3 and Figure 7).

## MATERIALS AND METHODS

### Microarray data

The gene expression profile GSE20347 was downloaded from GEO database including 17 EC samples and their paired adjacent non-cancerous samples. Platform information was GPL571 [HG-U133A_2] Affymetrix Human Genome U133A 2.0 Array.

### Data preprocessing

The probe-level data in CEL files were converted into expression value matrix by GCBI online platform analysis in the following link: www.gcbi.com.cn. Robust Multi-chip Average (RMA), including background-corrected, normalization and summary, was used to compute the expression value [22]. Quality control of gene expression data was performed using gene-specific probe. The median of NUSE value of each chip was applied to evaluate the feasibility of this design and the reliability of these analysis results. We further assessed the change rule of every probe set in the experiment by computation of RLE [23]. And the unified standard criterion for every sample was as follows: (1-0.2) < median < (1 + 0.2) and (−0.25) < median {RLE} < (0.25). The chips that did not meet this criterion were rejected. Data distribution was presented as box graph. Probe set annotation mainly referred the new version annotation files that were download on affymetrix official website (http://www.affymetrix.com/support/technical/annotationfilesmain.affx) and the probes without annotation were filtered.

### DEGs screening and hierarchical cluster analysis

First, we padded the signal values for low abundance genes. That was to say the signal value low than log3 were filled with log3. The no variant genes (same expression value) and low variant genes were filtered. GCBI filtered these genes whose detected percentage was below 50% in the whole expression profile. Then, DEGs between EC and normal tissues were identified with the FC method (no less than 3 biological replicates each group) and the Significance Analysis of Microarrays (SAM) method (no less than3 biological replicates each group) [24]. q value was calculated to control the FDR. The genes changed for more than 2 times in gene expression, P value < 0.05 and q value < 0.05 were selected as DEGs. Besides, hierarchy cluster analysis was performed and cluster dendrogram was constructed to ensure good characterizations of screened DEGs between EC and normal tissues [25]. In hierarchical cluster analysis, Pearson correlation was used to calculate the correlation between the genes and samples.

### Functional enrichment analysis

Functional analysis of DEGs was carried out by the Gene Ontology project (http://www.geneontology.org) on the basis of biological process [26]. The Fisher's exact test was used to classify the GO category, and FDR was calculated to correct the p-value. P value < 0.05 and FDR < 0.05 were used as a threshold to select significant GO categories. Besides, we calculated the enrichment score to access the enrichment level for per GO category. Pathway analysis was used to identify the significant pathways that DEGs participated in according to the KEGG database [27]. Fisher exact test and Benjamini-Hochberg step-up were used to calculated P value and FDR of per pathway. Those of P value < 0.05 and FDR < 0.05 represented the significant ones. The enrichment score was calculated to access the enrichment level for per pathway.

### Pathway-net analysis of the significant pathways

Pathway-net analysis, the interaction network of the significant pathways of DEGs, was built according to the interaction among pathways of the KEGG database to find out the relationship among the significant pathways directly and systemically. Each pathway in the network was measured by counting its number of upstream and downstream pathways, which were shown as in-degree and out-degree. A higher degree of a pathway indicated that it regulated or was regulated by other pathways, implying a more important role in the signaling network. It could summarize the pathway interaction of differential expression genes and found out the reason why certain pathway was activated [28].

### Signal-net analysis of the significant DEGs

Signal-net of the significant DEGs was constructed according to their betweenness centrality. The matrix of genes expression values was built up on the data of the interaction database from KEGG. Network was presented as graphs, where nodes were mainly genes and edges represented relation types between the nodes, e.g. activation or inhibition. The “betweenness” was defined as the signal transduction centrality of a gene in the gene network. A higher betweenness value of a gene implied a greater ability to mediate signal transduction.

### TCGA database analysis

TGCA database was derived from UCSC Cancer Browser (https://genome-cancer.ucsc.edu). Gene expression based on TCGA RNA-Seq data were shown as mean ± standard error of the mean (SEM) of triplicate determinants. The student's t-test was used for analyzing differences between independent data sets with normal distribution. OS and PFS analysis of EC patients with high and low levels of IL8 and CXCR7 based on TCGA RNA-Seq data set were shown by using a Kaplan-Meier survival plot. We used Kaplan-Meier curves to present the prognosis of the high and low groups. The Wilcoxon log-rank test was then conducted on the Kaplan-Meier curves to detect the survival difference between these two groups. All survival analysis was conducted using the R package Survival.

### Patients and tumor samples

For RT-PCR and survival analysis, 30 pairs of frozen EC and their adjacent normal tissue specimens were collected from patients with EC that were diagnosed from 2010 to 2011 at the Department of Thoracic Surgery of the First Affiliated Hospital of Zhengzhou University (Zhengzhou, China). For IHC analysis, The other 22 pairs of EC and their adjacent normal tissue specimens were freshly collected from patients with EC that were diagnosed from May 2017 to July 2017 at the Department of Thoracic Surgery of the First Affiliated Hospital of Zhengzhou University. No chemotherapy, radiotherapy, or other therapy was performed prior to entry into the study. Samples used in this study were approved by local ethics committees, and informed consent was obtained from each patient with available follow-up information.

### RNA extraction and RT-PCR

Total RNA was extracted from EC tissue specimens by TRIzol reagent (Invitrogen, Carlsbad, CA, USA) according to the manufacturer's instructions. The purity and concentration of RNA were detected using NanoDrop 2000 (Thermo Scientific, Waltham, USA). First-strand cDNA was synthesized from 1 μg of total RNA using PrimeScript™ RT reagent Kit with gDNA Eraser (TaKaRa, Dalian, China). The RT-PCR was performed using SYBR Premix Ex Taq II (TaKaRa) in Agilent Mx3005P. Three independent experiments were performed to analyze relative gene expression and each sample was tested in triplicate. Glyceraldehyde-3-phosphate dehydrogenase (GAPDH) was used for normalization of data. Primers used were *IL8*, 5′-TTTTGCCA AGGAGTGCTAAAGA-3′ (forward) and 5′-AACCCTC TGCACCCAGTTTTC-3′ (reverse), *CXCR7*, 5′-TCTG CATCTCTTCGACTACTCA-3′ (forward) and 5′-GTAGA GCAGGACGCTTTTGTT-3′ (reverse), and *GAPDH*, 5′-AGGAGCGAGATCCCTCCAAAAT-3′ (forward) and 5′-GGCTGTTGTCATACTTCTCATGG-3′ (reverse).

### IHC

IHC was performed according to standard protocols. Expression levels of IL8 (Abcam, Cambridge, UK, 1:1000) and CXCR7 (Abcam, Cambridge, UK, 1:800) were detected using IHC. All sections were assessed at 20× magnification by one pathologist and two experienced observers, and visualized under a microscope (Olympus, Japan). Staining was evaluated based on intensity (negative = 0; weak = 1; moderate = 2; and high = 3) of immunostaining and density (0% = 0; 1–40% = 1; 41–75% = 2; > 76% = 3) of positive tumor cells. The final immunoreactivity score of each sample was acquired by multiplying the intensity and density scores.

## CONCLUSIONS

In summary, our results provide a comprehensive bioinformatics analysis of genes and pathways which may be involved in the progression of EC. Total 1348 DEGs were achieved, and pathway-net and signal-net of these DEGs were constructed. GO and KEGG pathway enrichment analysis showed that DEGs mainly participated in the process of cell adhesion, cell proliferation, survival, invasion, metastasis and angiogenesis. Pathway-net and signal-net analysis revealed that the aberrant expression of MAPK signaling pathway, PI3K-Akt signaling pathway, pathways in cancer, pathways on cell adhesion, p53 signaling pathway, PLCD1 and PTK2 was extensively implicated with EC occurrence and development. Furthermore, we predicted the increased level of IL8 and CXCR7 played a prominent role in the pathogenesis of EC. The central pathways and significant genes identified by us can be used to distinguish EC samples from normal specimens. Our discovery may be of vital importance for investigating the complex interacting mechanisms underlying EC carcinogenesis and designing specific treatments for patients with EC, particularly the immunotherapy.

## SUPPLEMENTARY MATERIALS FIGURES


