# Correction: Gene function analysis and underlying mechanism of esophagus cancer based on microarray gene expression profiling

**DOI:** 10.18632/oncotarget.27371

**Published:** 2020-03-03

**Authors:** Ying Yue, Mengjia Song, Yamin Qiao, Pupu Li, Yiqiang Yuan, Jingyao Lian, Suying Wang, Yi Zhang

**Affiliations:** ^1^ Biotherapy Center, The First Affiliated Hospital of Zhengzhou University, Zhengzhou, Henan 450052, China; ^2^ Department of Oncology, The First Affiliated Hospital of Zhengzhou University, Zhengzhou, Henan 450052, China; ^3^ The No.7. People’s Hospital of Zhengzhou, Zhengzhou, Henan 450016, China; ^4^ Clinical Laboratory, Hebi People’s Hospital, Hebi 458030, China; ^5^ School of Life Sciences, Zhengzhou University, Zhengzhou, Henan 450001, China; ^6^ Key Laboratory for Tumor Immunology and Biotherapy of Henan Province, Zhengzhou, Henan 450052, China

**This article has been corrected:** Due to errors during figure assembly, the representative image of normal tissues in Figure 7E control group is incorrect. The correct Figure 7, obtained using the authors’ original data, is shown below. The authors declare that these corrections do not change the results or conclusions of this paper.

Original article: Oncotarget. 2017; 8:105222–105237. 105222-105237. https://doi.org/10.18632/oncotarget.22160

**Figure 7:**
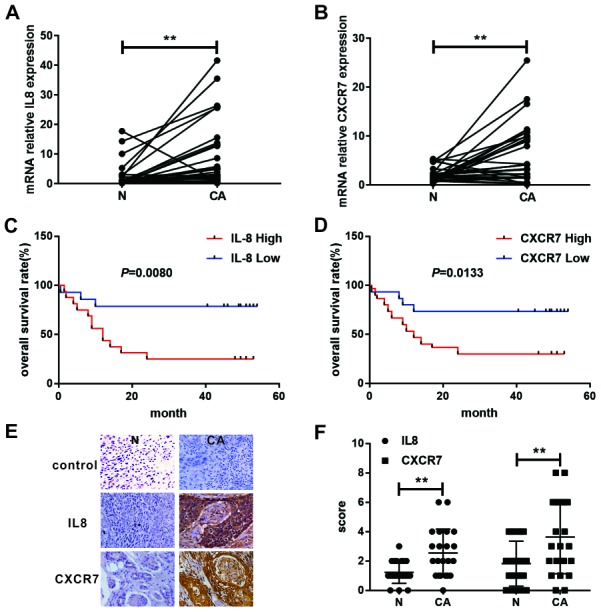
Expression and survival significance of IL8 and CXCR7 in patients with EC.

(**A**) IL8 mRNA relative expression in cancerous and their paired adjacent non-cancerous tissues in 30 patients with EC were analyzed by RT-PCR. (**B**) CXCR7 mRNA relative expression in cancerous and their paired adjacent normal tissues in 30 patients with EC were analyzed by RT-PCR. Kaplan-Meier survival curve for 30 EC patients with high (n=15) and low (n=15) IL8 (**C**) and CXCR7 (**D**) expression (RT-PCR analysis). (**E**) IL8 and CXCR7 expression in cancerous and paired adjacent normal tissue of EC were analyzed by IHC. (**F**) The final immunoreactivity score of IL8 and CXCR7 expression in 22 paired specimens was shown as statistical graph. (^**^ *P* < 0.01; N, non-cancerous tissue; CA, cancerous tissue).

