# Supplementary material for: Gene function analysis and underlying mechanism of esophagus cancer based on microarray gene expression profiling
Source: Oncotarget. 2017 Oct 30;8(62):105222–37. doi: 10.18632/oncotarget.22160 (PMC5739633; doi:10.18632/oncotarget.22160)
Supplement: Supplementary file 1 [file oncotarget-08-105222-s001.pdf]

Gene function analysis and underlying mechanism of esophagus cancer based on microarray gene expression profiling

SUPPLEMENTARY MATERIALS

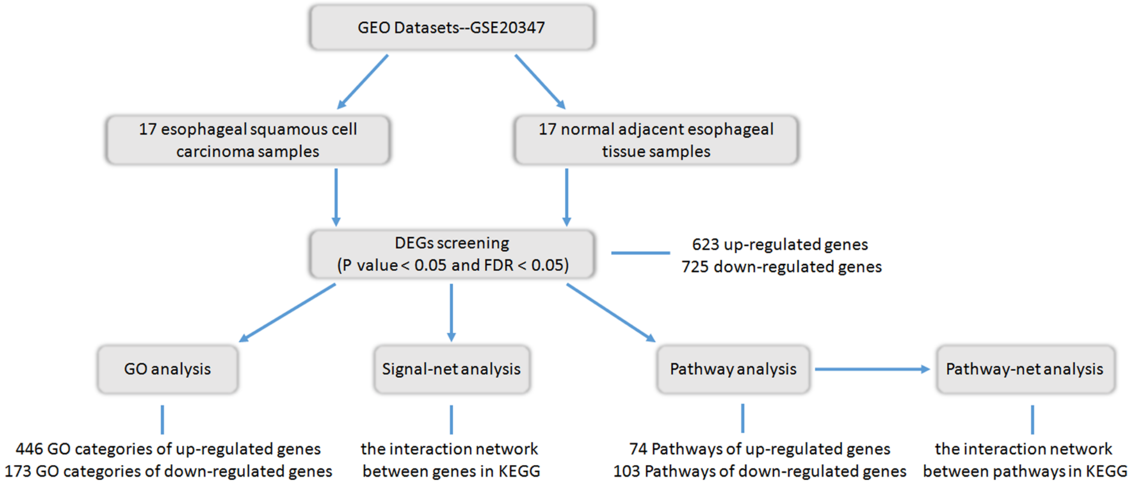

Supplementary Figure 1: The entire bioinformatic workflow. Gene expression profile GSE20347 was downloaded from GEO database, which contained 17 ESCC samples and their paired adjacent non-cancerous samples. Firstly, the differential expression between cancer and normal tissues was analyzed, and 623 up-regulated genes and 725 down-regulated genes were selected as DEGs by P value < 0.05 and FDR value < 0.05. Then, DEGs were applied to GO analysis and pathway analysis to identify function and regulatory pathways they participated in according to KEGG. 446 GO categories and 173 GO categories were found in up-regulated and down-regulated DEGs. 74 pathways and 103 pathways were found in up-regulated and down-regulated DEGs. Further pathway-net analysis was performed to build the interaction network between the pathways in KEGG. Signal-net analysis was established to clarify the interaction network between DEGs in KEGG.

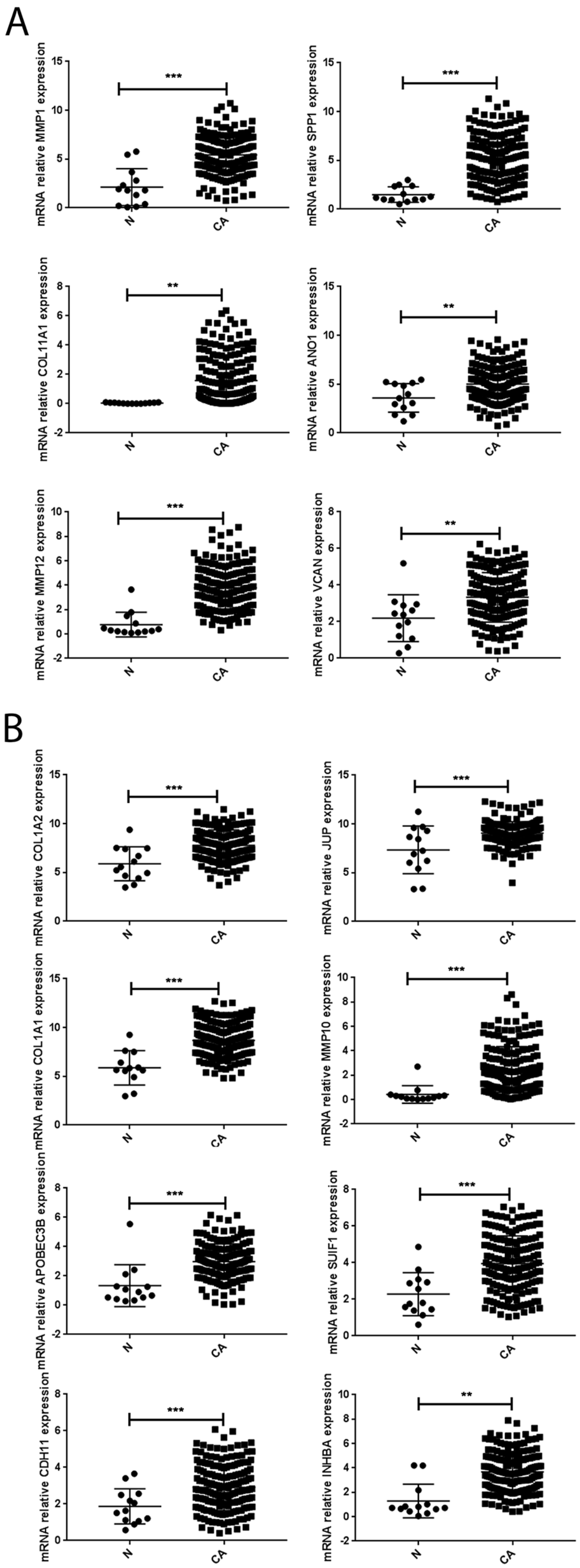

Supplementary Figure 2: Gene mRNA expression in TCGA database. (A) MMP1, COL11A1, SPP1, ANO1, MMP12, VCAN. **(B)** COL1A2, COL1A1, APOBEC3B, CDH11, JUP, MMP10, SUIF1, INHBA. $^{**}P < 0.01$, $^{***}P < 0.001$.

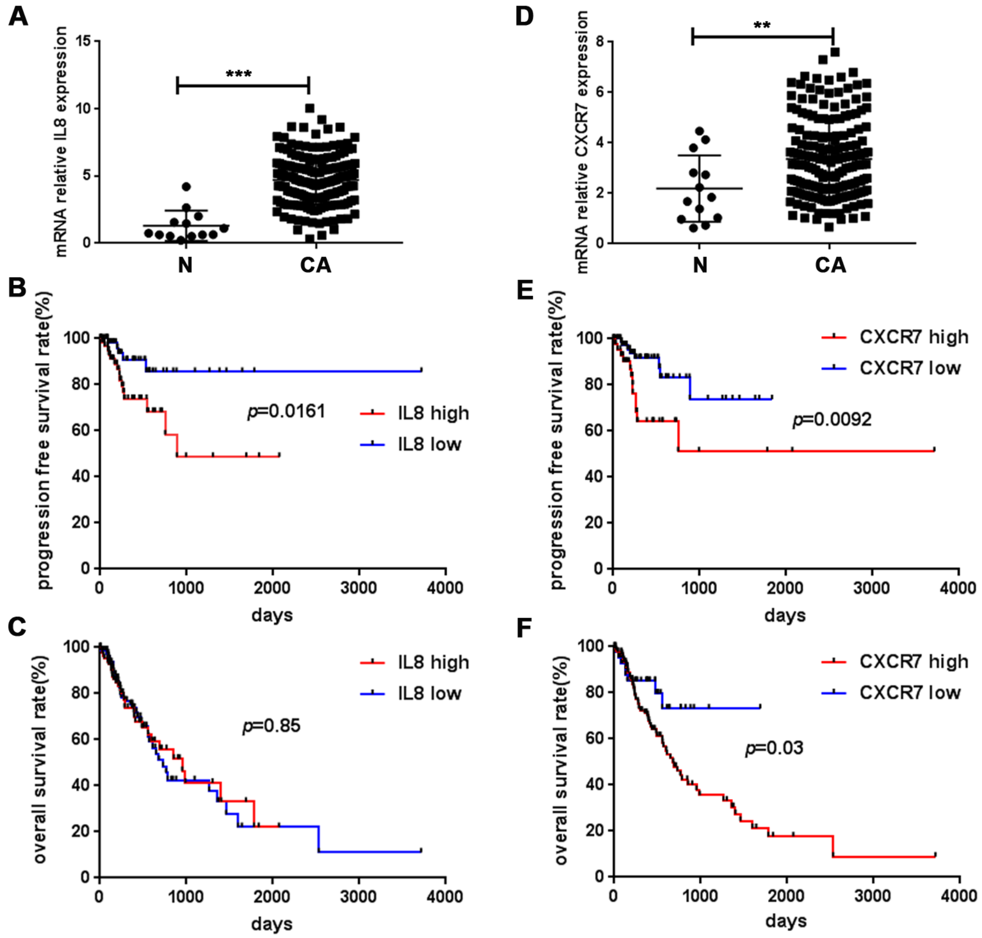

Supplementary Figure 3: Expression and survival curve of IL8 and CXCR7 in TCGA database. **(A)** IL8 mRNA expression in EC and normal tissues. **(B)** IL8 expression negatively correlated with progression-free survival. **(C)** IL8 expression did not correlate with overall survival. **(D)** CXCR7 mRNA expression in EC and normal tissues. **(E)** CXCR7 expression negatively correlated with progression-free survival. **(F)** CXCR7 expression negatively correlated with overall survival. $^{**}P < 0.01$, $^{***}P < 0.001$
